# Quantitative imaging of the complexity in liquid bubbles’ evolution reveals the dynamics of film retraction

**DOI:** 10.1038/s41377-019-0131-4

**Published:** 2019-01-30

**Authors:** Biagio Mandracchia, Zhe Wang, Vincenzo Ferraro, Massimiliano Maria Villone, Ernesto Di Maio, Pier Luca Maffettone, Pietro Ferraro

**Affiliations:** 1 CNR-ISASI, Istituto di Scienze Applicate e Sistemi Intelligenti “E. Caianiello” del CNR, Via Campi Flegrei 34, 80078 Pozzuoli, Napoli Italy; 2 College of Applied Sciences, Beijing University of Technology, 100124 Beijing, China; 3 Dipartimento di Ingegneria Chimica, dei Materiali e della Produzione Industriale, Università di Napoli Federico II, Piazzale Tecchio 80, 80125 Napoli, Italy

**Keywords:** Applied optics, Imaging and sensing

## Abstract

The dynamics and stability of thin liquid films have fascinated scientists over many decades. Thin film flows are central to numerous areas of engineering, geophysics, and biophysics and occur over a wide range of lengths, velocities, and liquid property scales. In spite of many significant developments in this area, we still lack appropriate quantitative experimental tools with the spatial and temporal resolution necessary for a comprehensive study of film evolution. We propose tackling this problem with a holographic technique that combines quantitative phase imaging with a custom setup designed to form and manipulate bubbles. The results, gathered on a model aqueous polymeric solution, provide unparalleled insight into bubble dynamics through the combination of a full-field thickness estimation, three-dimensional imaging, and a fast acquisition time. The unprecedented level of detail offered by the proposed methodology will promote a deeper understanding of the underlying physics of thin film dynamics.

## Thin liquid films: Seeing bubbles in a better light

A procedure for imaging the complex fluid dynamics in bubbles could greatly assist efforts to understand and exploit thin liquid films in applications ranging through medicine, industrial chemistry and engineering. Thin liquid films are ubiquitous in nature, found in such varied systems as soap bubbles, biological membranes, detergents, oils, insulation, foods and geological magma. Researchers in Italy led by Biagio Mandracchia at the Institute of Applied Science and Intelligent Systems in Naples, devised a novel holographic phase imaging technique to watch bubbles as they form, develop, burst and retract. The researchers built customized apparatus to create and manipulate the bubbles. The unprecedented level of detail being revealed offers deeper understanding of the physics underlying thin film behavior. Insights into the complex fluid dynamics within bubbles could advance thin film technology for many applications.

## Introduction

Thin liquid films, such as soap bubbles, are ubiquitous in nature and technology. Biological vesicles, magma bubbles, insulating and food foams, detergents, and oil foams all share most of the physics, chemistry, and engineering of bubble formation and evolution^1,2^. Studying these films is also important since they mediate a wide range of transport processes, encompassing applications from nanotechnology to biology^3–5^. These films may display unusual dynamics featuring the formation of regular or chaotic structures, periodic waves, shocks, fronts, and “fingering” phenomena^6^. The entire research area is currently thriving with new discoveries and applications, particularly techniques for measuring both the long-range thickness mapping and its fast acquisition on evolving thin films. In fact, the measurement of the thin film thickness evolution as a consequence of manipulation, drainage, and rupture is key to understanding such behaviors^7–9^.

Currently, different techniques for quantitative phase imaging (QPI) are used to measure the thickness of transparent three-dimensional (3D) objects with one dimension thinner than the other two (films)^10,11^. In particular, interferometry is routinely used for the study of thin fluid films and surface topology, using both monochromatic and white light^12,13^. Interferometry measures the intensity of fringes produced by the interference of light reflected at the two interfaces of a thin film. Such intensity depends on the wavelength of light, the refractive index of the sample, and the thickness of the material. These techniques can be divided into two families, characterized by point-like or full-field inspection^9^. The first family of techniques measures the thickness in a very restricted area of the film’s surface. Early studies used a photomultiplier to precisely measure the equilibrium thickness of soap films contained in a special cell, designed to isolate a thin film of liquid^14^. Modernized versions of this setup are currently used by several research groups^15,16^. Conversely, the full-field techniques measure the thickness across the entire surface of the film throughout the experiment^17,18^. Even though these systems can determine the film thickness with a resolution of a few nanometers, they lack the (lateral) spatial or temporal resolution necessary to follow the complex dynamics of an evolving thin liquid film.

In this study, we propose the adoption of a setup for the study of thin film dynamics based on off-axis digital holography (DH). Holographic microscopes are interferometers that allow for a pseudo-3D reconstruction of objects captured out of the best focal plane. This feature adds flexibility to the experimental procedure and in turn has kindled the spreading of DH beyond the field of metrology, from non-destructive testing for industry to label-free imaging of biological samples^19–22^. DH can accurately determine the phase and amplitude by means of dense carrier fringes down to fractions of the illumination wavelength. A benefit of digital holography with carrier fringes is that, unlike some other QPI techniques, e.g., phase shift interferometry^23^, the necessary information is completely gathered into a single frame, which is appropriate for high-speed data acquisition.

We report the measurement of the entire thickness distribution over an aqueous polymeric thin film solution during the formation of a bubble under non-ideal conditions, where several film thicknesses are simultaneously present in the film. Based on these data, the variation range and variation trend of the film thickness map are accurately measured, from the formation to the inflation and the bubble rupture. In particular, during the bubble growth, the location of the bubble surface changes continuously so that an imaging system in which the focusing of the image can be retrieved ex post from the experimental recordings is required. DH allows such a refocus of the sample by numerical processing of the recorded holograms^24^. In this way, it is possible to follow the position of the film surface a posteriori during bubble formation.

## Results

### Holographic thickness mapping for liquid films

The experimental setup was designed by embedding a custom setup to form and manipulate a thin liquid film within an off-axis Mach–Zehnder interferometer (see Figure S1a). The films are formed on top of a metal pipe with an internal diameter of 18 mm and a side inlet connected to a syringe pump (see Figure S1b-c). As a model system, we studied the temporal evolution of the thickness profile of bubbles formed from a film made of an aqueous solution of maple syrup and 0.05 wt% polyacrylamide (PA). The bubbles were inflated by pumping air from the side inlet of the pipe at a flow rate φ = 0.015 mL/s^25^. DH in off-axis geometry is based on the classic holography principle, with the difference being that the hologram recording is performed by a digital camera and transmitted to a computer, and the subsequent reconstruction of the holographic image is performed numerically (see Figure S1d-g). In DH, the interferometric acquisition system can only measure the phase modulo-2π, commonly referred to as the wrapped phase. To recover the absolute phase, and then the thickness profile, we used the Phase Unwrapping Max-flow/min-cut (PUMA) method^26^. The PUMA method provides an exact energy minimization algorithm given the assumption that the difference between adjacent pixels is smaller than π rad. From the experimental point of view, this leads us to ensure that we have a good sampling of the observed area in order to assume that the thickness changes are sufficiently smooth in comparison to the fringe sampling, and no phase jumps are missed.

**Fig. 1:**
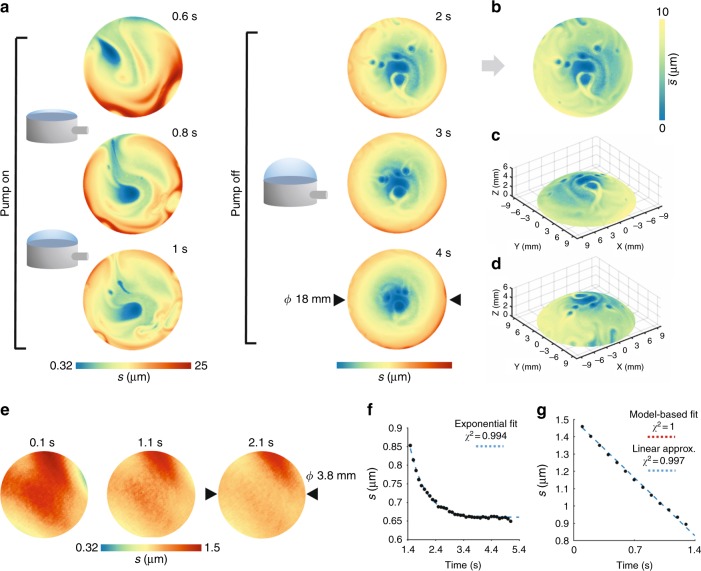
Holographic thickness mapping during inflation and drainage. **a** Evolution of the film thickness during bubble inflation (left) and drainage (right). The thickness values were obtained by holographic measurements, where the refractive index of the sample was known to be 1.47. During the experiment, the film was inflated for 2 s and then allowed to drain naturally until rupture. **b** Corrected map of the film thickness. Assuming that the bubble surface can be approximated by a spherical cap, it is possible to retrieve the film thickness in the radial direction. Three-dimensional depictions of the radial thickness map are shown in (**c**) and (**d**). **e** Drainage and film thinning at the center of the bubble. Thickness maps of the center of a bubble obtained by digital holography. The bubble was allowed to grow for 14 s. Afterwards, the pump was turned off and the fluid was allowed to drain naturally until rupture. Scale bar 1 mm. **f** Plot of the thickness as a function of time during bubble blowing. **g** Plot of the thickness as a function of time during gravitational drainage

Once retrieved, the absolute phase gives a measurement of the optical path length experienced by the laser beam, which is equal to the thickness of the film multiplied by its refractive index. Thus, knowing the refractive index of the solution bunches used in the experiments (see Supplementary Information), we can easily map the evolution of the film thickness during the bubble growth and drainage (see Fig. 1a and Supplementary Videos 1 and 2).

Finally, DH acquisitions are pseudo-3D representations of the optical thickness of the sample. This means that the measured thickness profile, *s*, is a projection on the image plane of the three-dimensional structure of the sample (see Figure S3). However, the thickness normal to the bubble surface, $\bar s$, can be retrieved by geometric considerations (see Fig. 1b–d and Supplementary Video 3). It is worth noting that near the center, the two values are almost identical. For example, within 1.3 mm from the center, the estimated relative error is less than 1% (see Figure S4).

### Film thinning and bubble growth

The shape of the bubble in our system is mainly controlled by the volumetric air flow, $\varphi$, set by the pump, and, if $\varphi$ is constant, the volume of the bubble grows linearly in time:

1 $$V_{\mathrm {bubble}} = \varphi t$$

Considering the bubble as a spherical cap of height *h* and basal radius *a*, we can rewrite the previous equation as:

2 $$\frac{\pi }{6}h\left( {3a^2 + h^2} \right) = \varphi t$$

The geometric parameters of the bubble can then be fully controlled by the pump.

To study the film thinning due to the gravitational drainage of the fluid along the bubble surface, we adjusted the experimental parameters in order to maximize the bubble stability while approaching the hemispherical shape. We observed that reasonably stable bubbles could be formed by inflating air into the metal pipe with a relatively low flow of φ=0.015 mL/s. Nonetheless, we found it difficult to reach a perfect hemispherical shape of the bubble (*h*∼*a*). Furthermore, this configuration was impractical for the study of drainage towards the borders, as discussed in the previous section, so we decided to stop the pump at a height of approximately two-thirds of the basal radius (*h*∼2/3*a*).

Bubbles were observed from the top and from the side. The top view was recorded by a CCD (charge-coupled device) camera at a maximum frame rate of 60 Hz. The side view was recorded by a CMOS (complementary metal-oxide semiconductor) camera (Apple Inc. iSight) at 30 Hz (see Supplementary Video 4). The experiments were conducted at 23 °C.

During inflation, *h* is a function of time and Eq. (2) can be rewritten as:

3 $$h\left( t \right)\left( {h^2\left( t \right) + 3a^2} \right) = \frac{{6\varphi }}{\pi }t$$

From which we can derive the following formula:

4 $$h\left( t \right) = \frac{{\root {3} \of {{\sqrt {4a^6 + b^2t} + bt}}}}{{\root {3} \of {2}}} - \frac{{\root {3} \of {2}a^2}}{{\root {3} \of {{\sqrt {4a^6 + b^2t} + bt}}}}$$

with $$b = \frac{{6\varphi }}{\pi }.$$

A good fit of the experimental data is given by a first-order approximation of Eq. (4) (see Figure S1h):

5 $$h\left( t \right) = \gamma \root {3} \of {{1 + \beta t}} - \frac{\gamma }{{\root {3} \of {{1 + \beta t}}}}$$

The thickness maps in Fig. 1e show an accumulation of the fluid in the central region, when the bubble is still flat (prior to pump starting, *t* = 0.1 s). During inflation, it is possible to observe a gradual thinning at the center that slowly continues when pumping is stopped. This process is the consequence of the gravitational drainage of fluid from the top towards the rim of the bubble.

Gravitational drainage causes the film thickness to decay exponentially with time^27^. Now, recalling that in the center, $s = \bar s$, we have that:

6 $$s = s_0e^{ - \frac{t}{\tau }}$$

where

7 $$\tau = \frac{\alpha }{h};\alpha = \frac{\mu }{{\rho g}}$$

where *μ* is the viscosity, *ρ* is the liquid density, *g* is the gravitational acceleration, and $s_0$ is the initial thickness, see Fig. 1f.

During inflation, the film thickness of the bubble has a more complicated dependence with time. Indeed, the drainage is concurrent with the film stretching as a consequence of the increase in the bubble surface. However, the experimental data can also be satisfactorily approximated by a linear function (see Fig. 1g):

8 $$s = s_0\left( {1 - \beta t} \right)$$

### Fluid drainage and convection

**Fig. 2:**
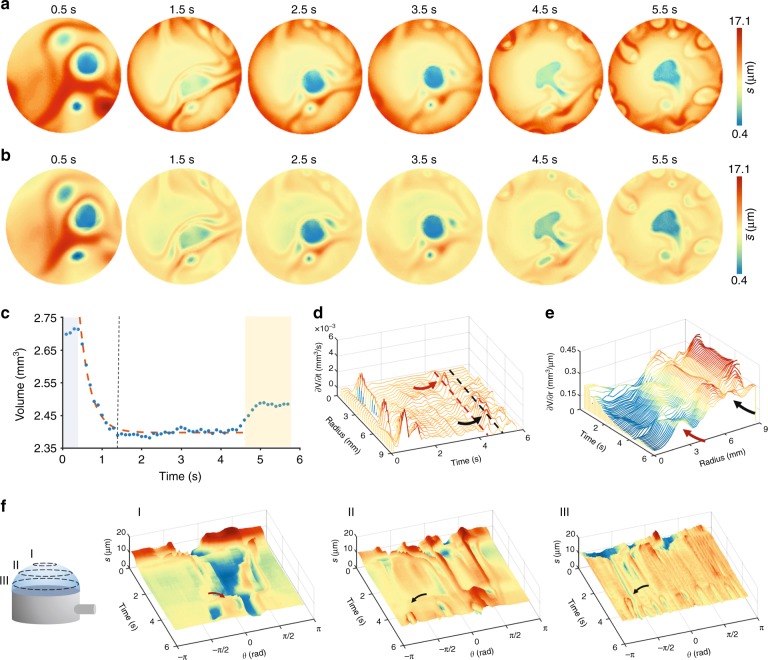
Fluid drainage. Thickness maps of the entire bubble surface before (**a**) and after (**b**) curvature correction. At first, the fluid accumulates in the center with no particular ordering. It is possible to see very thin areas in the film randomly positioned. While the bubble is growing, however, most of the fluid is drained towards the edges and the thin films move to the center. **c** Plot of the volume as a function of time. After an initial stasis time, the mass drainage appears to follow the expected exponential behavior. However, just before the rupture time, the volume starts to increase once again. The derivatives along the time (**d**) and radius (**e**) show that this volume increase moves from the rim toward the center (see black arrows). **f** Thickness change vs time and azimuthal angle (*θ*) at three different distances from the bubble center, namely (I) 3 mm, (II) 6 mm, and (III) 9 mm. It is possible to observe some amounts of mass flowing back from the rim to the center

The continuous drainage towards the borders causes a decrease in the mass of the fluid with time. This is directly proportional to the volume of the film layer: $V = M{\mathrm{/}}\rho$. Ideally, if the fluids were perfectly homogeneous, we would expect the drainage to be radial. This means that the thickness of the film does not depend on the polar angle but only on the latitude. This assumption fails for real films, where some level of inhomogeneity or asymmetry is present in the system and gives rise to various phenomena, such as convection of the fluid inside the film. As expected, the center of the bubble tends to become thinner the larger the bubble becomes (Fig. 2a, b and Supplementary Video 5). Nonetheless, this phenomenon is not homogenous. At the same time, it can be noted that this change in thickness does not happen uniformly, but it seems to be related to a momentary rearrangement of the fluid across the surface.

After an initial stasis period (Fig. 2c, gray area), the bubble volume drops with time and follows the expected exponential decay (red dashed line). Surprisingly, after reaching a plateau value, the volume begins to grow again a few seconds before the rupture (see Fig. 2c, yellow area). A more detailed analysis reveals that this increase is related to a change in the drainage dynamics of the fluid (see Fig. 2d, e). It is possible to devise two different contributions to this inversion of the trend, one at the center of the bubble and the other close to the edge of the pipe. The first contribution is due to a relatively small increase in the thickness of the film around the center (Fig. 2d–f, red arrows). The second contribution is given by a steady in-flow of part of the fluid from the edge of the pipe back towards the center of the bubble (Fig. 2d–f, black arrows). This in-flow takes the form of a regular pattern which can be devised after 4.5 s. The regular patterns observed at the latest stage of the film evolution dynamics, depicted in Fig. 2b, have been observed elsewhere for vertical and horizontal thin films and are usually addressed to as “fingers” due to the Marangoni effect, Plateau-Rayleigh instability, and/or marginal regeneration. In the context of Plateau–Rayleigh instability, in a cylindrical flow with infinite length, the characteristic wavelength of the pattern is:^28^

9 $$L = \frac{s}{{4\pi }}\sqrt {2 + 3\sqrt 2 Oh}$$

where $Oh = \frac{\mu }{{\sqrt {2\gamma \rho s} }}$. In our case, we found *L*=2.2 mm, which is of the same order of magnitude of the experimental value $L_{\mathrm {exp}} = 2\pi R{\mathrm{/}}N$=5.4 mm, where *N* is the number of fingers on the image (*N* = 9) and *μ* is the viscosity. Furthermore, the motion of these patterns could be due to the marginal regeneration: along the edge of the film, where the film connects with the pipe, there is a “Plateau border” that has curved surfaces and a lower Laplace pressure than the central part of the film; thicker parts are bodily drawn into the border by the negative excess pressure, while the thinner film is pulled out of the border.

To give a plausible physical interpretation to the experimentally observed non-monotonic trend of bubble thickness shown in Fig. 2c, we performed a Finite Element numerical simulation of a system mimicking the experimental one. The mathematical model underlying the numerical simulation is created from the mass and momentum balance equations and the constitutive equation for the liquid film supplied with proper boundary and initial conditions (see the Supplementary Information for details). The constitutive parameters of the liquid have been derived from the rheological data of the fluid employed in the experiments (see the Supplementary Information).

The numerical temporal trend of the thickness at the center of the film *h*, normalized by its initial value *h*_0_, is reported in Figure S2. By comparing Fig. S2c and Fig. 2c, it is apparent that even if in the simulation a simplified system is considered, a good agreement holds between the experimental behavior of the volume of the bubble central portion and the simulated evolution of the film thickness, with an initial steep decrease while the bubble is inflated, an almost horizontal portion, and then an inversion of the trend, i.e., a thickening at the center of the bubble.

From the outcome of the numerical simulation, the latter can be ascribed to fluid drainage from the rim deposited on the pipe edge toward the center of the film due to the surface tension. Indeed, two opposite mechanisms act: during inflation, the film thins are at the center due to gravity and liquid adherence at the pipe wall, whereas surface tension makes the fluid move from the border to the center to minimize the film’s external surface. Since inflation is fast, at the beginning, the effects connected to it dominate; then, when inflation ends, the “reservoir” constituted by the rim “pumps” the liquid back, thus making the film thicken at the center.

**Fig. 3:**
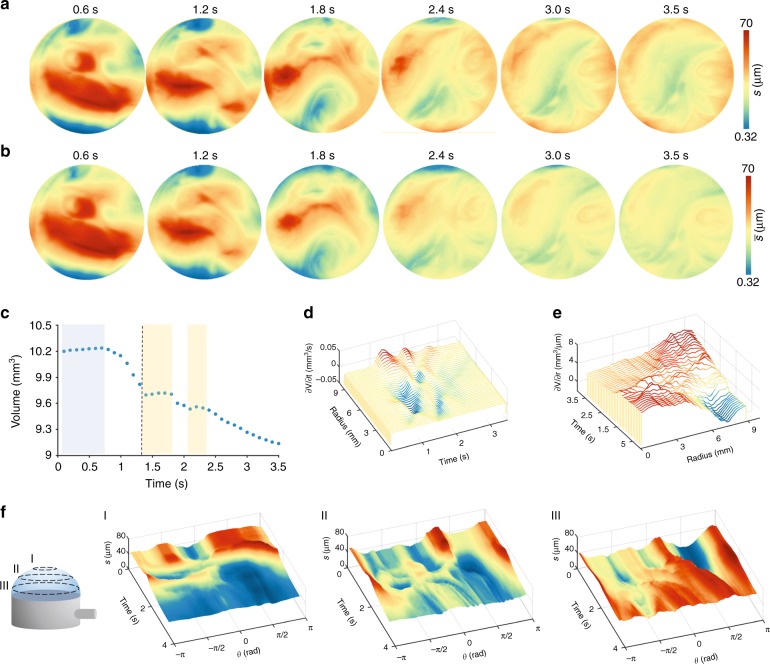
Complex fluid drainage. Thickness maps of the entire bubble surface before (**a**) and after (**b**) curvature correction. At first, most of the mass accumulates in the center. While the bubble is growing, however, the fluid is drained towards the edges. This process is chaotic and includes the presence of vortexes. **c** Plot of the volume as a function of time. The non-ideal nature of the drainage causes part of the fluid to come back towards the center of the bubble. This explains the presence of brief plateaus. Derivatives of the volume along time (**d**) and radius (**e**). **f** Thickness vs time and azimuthal angle (*θ*) at three different distances from the bubble center, namely (I) 3 mm, (II) 6 mm, and (III) 9 mm. The asymmetry of the process is especially clear in sections I and II

Figure 3a depicts a thin liquid film thickness evolution in a slightly different case in which the film is left for a long period of stasis before the inflation. In fact, thickening in the central part of the film is observed, due to sagging. Moreover, the topography of the film appears less homogenous than in the case of Fig. 2, which is further evidence of the thickness measurement accuracy of the proposed technique, particularly when inhomogeneities are present. In these cases, the mass of the fluid tends to accumulate at the center of the film before inflation begins (see Fig. 3a, b). However, it quickly drains towards the rim once the bubble begins to grow. In this case, the dynamics of the drainage process are not only far from ideal but, at certain moments, the entire process seems to stop (see Fig. 3c, yellow areas). On the other hand, the rate of volume drainage does not go to zero, nor in time nor along the radius (see Fig. 2d–f). This supports the fact that the rapid movement of a large quantity of mass creates some complex movement of the fluid at the rim, which could temporarily counterbalance the draining process (see Supplementary Video 6).

### Flow tracking

It is generally hard to describe a situation of complex motion such as the one depicted in Fig. 3. On the one hand, we have shown how it is possible to estimate the drift of the fluid and the dynamics of formation and dissolution of mass aggregates due to the presence of fluid vortexes. On the other hand, the assessment of the dynamics of liquid film rearrangement can be simplified following the displacement of particles dispersed in the fluid by holographic three-dimensional tracking.

**Fig. 4:**
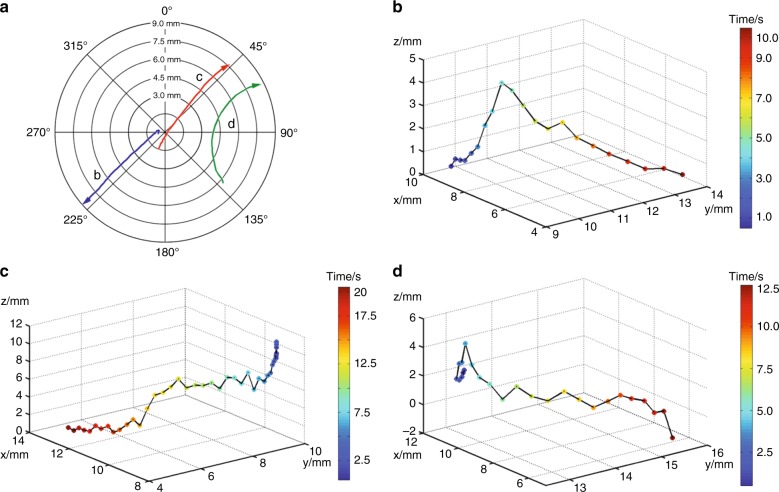
Three-dimensional (3D) tracking of poly(methyl methacrylate) (PMMA) particles diffusing in a liquid film. **a** Top view of the motion of three particles diffusing on the bubble surface. Their motion shows the presence of radial mass currents and vortexes. **b**–**d** Three-dimensional plot of the motion of each particle

We injected poly(methyl methacrylate) (PMMA) particles with a nominal diameter of 6 μm into the PA solution and tracked them in three dimensions via automated numerical refocusing (see Fig. 4). After holographic amplitude reconstruction, three particles were selected from all visible points, which followed different paths along the bubble surface. To effectively identify and assess the movement of these particles, we used the correlation recognition tracking method^29,30^.

All three particles have different trajectories and show non-trivial flows within the film. Indeed, it is possible to observe how they can have both radial and swirling motion. The observed speed of the process and its span in the third dimension make tracking the particles difficult using standard imaging techniques. Holographic 3D tracking, conversely, has proven suitable for these situations. This piece of information can be useful for not only analyzing the mass flow on a bubble film surface but also for following the arrangement of colloids inside the film.

### High-speed holographic imaging

The rupture of a bubble is a very fast process that requires the use of high-speed cameras to be observed. It can have very different dynamics, depending on the particular fluid or conditions of breakage^31^. One important parameter is the thickness of the opening rim and the possible presence of fluid droplets escaping from the film^25,32^.

To induce the rupture, we placed a needle on top of the metal pipe we used to grow the bubble. When the bubble reached an almost hemispherical shape, we gently lowered the needle until it was in contact with the film. To record the bubble rupture, we used a high-speed CMOS camera (Mikrotron, MC1310, 980 Hz).

**Fig. 5:**
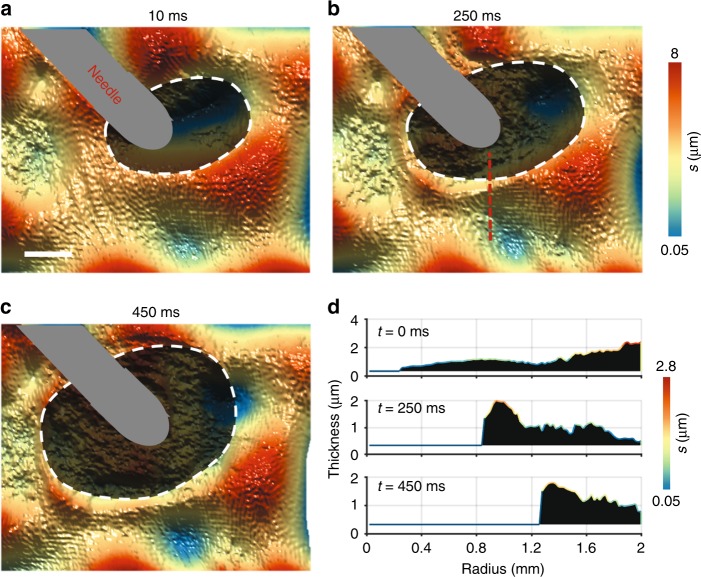
High-speed holographic imaging of bubble rupture. **a**–**c** Formation of the black spot around the needle tip (white dashed line). The black area indicates the phase noise related to the absence of light. The gray area indicates the position of the needle. Scale bar 1 mm. **d** Thickness profile at the rim of the black film (red dashed line). During retraction, the film tends to accumulate at the rim until rupture

Before the bubble rupture, it is possible to see a black film forming in correspondence with the tip of the needle (see Fig. 5a–c, white dashed line). The black film forms where the film thickness is half the illumination wavelength, when the local destructive interference cancels the light passing through. In holographic reconstructions, this local absence of light is associated with the generation of random values. This is why, in phase images, black spots correspond to areas of low signal-to-noise ratio. Finally, after approximately 453 ms, the boundary breaks and the bubble opens (see Supplementary Video 7).

High-speed holographic imaging can be useful for studying the mechanics of bubble rupture in deeper detail. Quantitative thickness mapping is essential for distinguishing the diverse profiles of the hole’s rim, which characterize the retraction behavior of fluids^33^. Moreover, when asymmetric breakage profiles are observed^34^, thickness mapping provides a link between the rupture path and the topography of the film.

For the first time, we observe the mass accumulation at the rupture edge during the film retraction, in accordance with the model proposed in ref. ^33^ (see Fig. 5d). During retraction, the film tends to accumulate at the rim, and then it becomes flatter during the last moments of the breakage. Also, the thinning process steadily continues and the black film rapidly expands around the needle. However, further analysis reveals that the boundary seems to move faster along the directions where the film is thinner (*θ* = 180° and 270°), probably following a least-resistance path (see Figure S3).

## Discussion

The study of thin films and bubble rupture is of great interest to industrial processes and life science. Indeed, foams as well as plasma membranes or vesicles can be modeled in a manner similar to soap films and bubbles. The nature and properties of such structures have been the subject of extensive studies and continue to be attentively investigated^35^.

The characteristics of these systems, representing the hardest characterization challenges, can be summarized as follows. First, they have fast and ever-changing dynamics, and hence real-time imaging systems and possibly fast recording devices should be used. Second, the film thickness varies from tens of micrometers to a few hundred nanometers. This depends strongly on the nature of the solution and on the experimental conditions utilized for film formation. Last, but still important, the bubble film is not uniform. This means that the bubble surface is a complex system and has a unique structure each time a new sample is prepared. The distribution of the polymer across the film changes every time and, even under the same pumping conditions, the time to rupture is not constant. Using an air flow of 0.015 mL/s, we saw this time change from 3 s to 10 s. It is likely that such a difference is due to both the initial bubble thickness and the evolution dynamics. This is why evaluating the film thickness based on geometric considerations is not sufficient; instead, a continuous and quantitative inspection is necessary.

In this work, the design and implementation of a setup for imaging the dynamics of thin bubbles is presented. Our setup is based on DH to obtain quantitative images of the sample film dynamics. Throughout the past few years, many methods based on interferometry have been proposed to measure the actual film thickness and to monitor the interfacial rheological properties of these systems. Differential interferometry methods have also been described in investigations of contact angles^36^ and bubble caps^37^. A method based on phase shift interferometry was developed for measurements on vertical films^38^. Other approaches based on resonant differential interferometry, fringe patterns from a dual-wavelength reflection, and speckle interferometry have also been reported^39,40^.

Compared with the abovementioned interferometric techniques, DH combines several advantages. First, the spatial resolution is limited only by the optics used, which is not true for methods that use color matching, where the thickness is measured at a few points and then interpolated over the entire image^9^. Second, it gives the full-field three-dimensional information of the sample, unlike techniques that use photomultipliers to have very fast measurements but only at one point^14^. Third, DH does not require multiple exposures and can be matched with high-speed cameras to measure rapidly changing features^23^, such as the rim of the hole formed by the rupture of the liquid film.

Due to the spatial resolution and fast, full-field measurement of the liquid film thickness, we proved that this technique has several novel features. In Fig. 3, we showed the time dependence of the film thickness on an evolving geometry of the bubble (in the past, the only way to measure the bubble’s thickness was without or after inflation). In Figs. 4 and 5, we showed the film volume’s evolution and observed that, for example, in the last few seconds, the average film thickness increases, although drainage towards the bottom would have suggested a monotonic reduction of the average thickness. This thickening is caused by mass fluxes from the border of the film through the center, as is shown in Fig. 2 using gradient plots. As such, this technique can be used to investigate phenomena such as tear spreading or coffee ring formation^3,9,41^, where Marangoni effects, drainage, and wetting concur with the thin film evolution.

In conclusion, we proposed an experimental setup that for the first time gathers all the features required to study the liquid thin film evolution. Nevertheless, this comes at the price of a more complex data analysis. However, there are now diverse resources available for both hologram reconstruction and data analysis, so that a custom code is seldom needed. Furthermore, the local thickness is calculated assuming a certain degree of continuity, i.e., step heights of less than half a wavelength. Even if the results do not show any contrary evidence and seem to be in agreement with the expected values, it can still be viewed as a limitation. This limitation can be overcome by changing the system used for the bubble formation, e.g., growing bubbles on top of a glass surface. Then again, future work should focus on the implementation of a robust optical solution, such as dual-wavelength DH. This method can considerably extend the dynamic range of phase detection, removing most of the issues related to phase wrapping.

The application of DH is not limited to the proposed configuration but could be adapted to different ones without difficulty. The metal pipe, for example, can be replaced by a quartz cuvette, which would be useful to study the formation of gas bubbles in a fluid^42^ (see Figure S4a). Alternatively, a configuration similar to the one proposed for phase shift interferometry can be used to study spherical bubbles pending from a nozzle^23^ (see Figure S4b). If controlling the volume is not essential, the bubbles could be grown on a glass substrate or a Petri dish^27^, as in Figure S4c. In this case, the illuminating beam could be slightly tilted in order to avoid illuminating the needle. In this way, it is possible to image the very first moments of the bubble rupture close to the tip of the needle. Finally, virtually all the systems currently used for the study of flat bubbles could be easily integrated into a holographic microscope^7,8,18^ (see Figure S4d).

## Materials and methods

### Experimental setup

The DH setup consisted of an off-axis Mach–Zehnder interferometer with a sample stage adapted for the control of bubble growth. The experimental setup is schematically shown in Figure S1a. The illumination source was a HeNe laser (λ = 632.8 nm). In the Mach–Zehnder interferometer, the laser beam is divided into two parts by a polarizing beam-splitter cube. The resulting beams are referred to as the object and reference beam. The object beam illuminates the sample from the top and forms the image on the camera. On the contrary, the reference beam goes towards the camera without passing through the sample. The two beams are collected by a second beam-splitter cube, which is slightly tilted so that the two beams overlap with a small angle. This angle controls the period of the interference fringes and can be adjusted according to the sampling requirements. The image of the sample is de-magnified by a factor of 0.25 with two lenses put in front of the camera (*f* = 200 mm and 50 mm). With an estimated maximum diameter of the circle of confusion of 0.4 mm, the depth of focus of the system is 8 mm.

### Bubble formation

The bubble growth was controlled using a custom metal pipe (see Figure S1b-c). The pipe had a diameter of 18 mm and a side inlet that was connected to a syringe pump (Harvard Apparatus). The rim of the top of the pipe was slightly grooved to maximize the contact surface with the bottom of the bubble. An aqueous solution of maple syrup (Maple Joe, Famille Michaud Apiculteur, Gan, France) and 0.05 wt% polyacrylamide (Saparan MG 500, The Dow Chemical Company, Midland, MI, USA) was used. Bubbles were created forming a film made of the solution on top of the pipe and placing the bottom on a glass Petri dish. The pipe was secured to the sample holder to prevent any possible movement during the measurements. Finally, a syringe pump (Harvard Apparatus, Model 22) was utilized to inflate the film and form a half bubble with a flow rate of 0.015 mL/s. A small amount of water was added onto the dish to avoid pumped-air leakage.

### Wavefront reconstruction

Digital holography in off-axis geometry is based on the classic holography principle, with the difference being that the hologram recording is performed by a digital camera and transmitted to a computer, and the subsequent reconstruction of the holographic image is performed numerically.

The recorded intensity *I*_*H*_*(x*_*H*,_*y*_*H*_*)* at the hologram plane is the square module of the amplitude superposition of the object and reference waves. It is given by:

10 $$	I_H\left( {x_H,y_H} \right) =\\ 	\left| {O_0\left( {x_H,y_H} \right)} \right|^2 + \left| {R_0} \right|^2 + O_0^ \ast \left( {x_H,y_H} \right)R_0 + O_0\left( {x_H,y_H} \right)R_0^ \ast$$

The phase information of the hologram is provided only by the last two terms, which are filtered and centered in the Fourier space. We reconstructed the holograms by numerically propagating the optical field along the *z* direction using the angular spectrum method. If *E(x,y;0)* is the wavefront at plane *z* = 0, the angular spectrum *A(ξ,η;0)=F{E(x,y;0)}* at this plane is obtained by taking the Fourier transform, where *F{}* denotes the Fourier transform; *ξ* and *η* are the corresponding spatial frequencies of *x* and *y* directions, respectively; and *z* is the propagation direction of the object wave. The new angular spectrum A at plane *z* = *d* is calculated from *A(ξ,η;0)* as:

11 $${A\left( {\xi ,\eta ;d} \right) = A\left( {\xi ,\eta ;0} \right) \cdot {\mathrm{exp}}\left\{ {j\frac{{2\pi d}}{\lambda }\left[ {1 - \left( {\lambda \xi } \right)^2 - \left( {\lambda \eta } \right)^2} \right]^2} \right\}}$$

The reconstructed complex wavefront at plane *z* *=* *d* is found by taking the inverse Fourier transform as

12 $$E\left( {x,y;d} \right) = F^{ - 1}\left\{ {A\left( {\xi ,\eta ;d} \right)} \right\}$$

where *F*^*−1*^*{}* denotes the inverse Fourier transform. The intensity image *I(x,y;d)* and phase image *φ(x,y;d)* are simultaneously obtained from a single digital hologram by calculating the square module of the amplitude and the argument of the reconstructed complex wavefront:

13 $$I\left( {x,y;d} \right) = \left| {E\left( {x,y;d} \right)} \right|^2$$

14 $$\phi \left( {x,y;d} \right) = {\mathrm{arctan}}\left( {\frac{{Im\left[ {E\left( {x,y;d} \right)} \right]}}{{Re\left[ {E\left( {x,y;d} \right)} \right]}}} \right)$$

The workflow of the numerical reconstruction is shown in Figure S1d.

From the experimental data, we observed that for each frame’s spectrum, the +1 order center changes with the bubble growth. We assumed that this phenomenon was due to the change in the bubble’s surface. The bubble surface could be seen as a lens, twisting the object beam and slightly changing the off-axis angle during growth. Therefore, if we use the same filtering window for each frame of the holographic video, in the final phase result, we would obtain a random phase distortion, which would greatly affect the phase measurement accuracy. This issue was addressed using an automatic filtering algorithm during the holographic video reconstruction. This algorithm simply scans the Fourier spectrum for the maximum of the +1 diffraction order and centers the filter accordingly.

### Thickness estimation

In DH, thickness estimation is directly related to the accuracy of the absolute phase recovery. Indeed, an interferometric acquisition system can only measure the phase modulo-2π, commonly referred to as the wrapped phase. Formally, we have $\phi \left( {x,y;d} \right) = \psi \left( {x,y;d} \right) + 2k\pi$, where *ϕ* is the absolute phase value, *ψ* is the wrapped phase, i.e., the measured value, and $k \in {\Bbb Z}$ is an integer accounting for the number of 2π multiples.

The main task of a phase unwrapping algorithm is the choice where the phase of the field should be shifted. In real experimental conditions, such a choice is often complicated by a phase noise, which can lead to erroneous phase unwrapping shifts. Since the phase noise often has a higher frequency than the desired signal, initial filtering of the wrapped phase field is the easiest and most intuitive way to simplify unwrapping^43^.

In this work, we used the PUMA method^26^. The PUMA method provides an exact energy minimization algorithm under the assumption that the difference between adjacent pixels is smaller than π rad. From an experimental point of view, this leads us to assume that the film thickness changes are smooth enough to be well sampled by the camera pixel. This assumption can be aided by changing the magnification of the system according to the homogeneity of the sample. A good sampling of the observed area is very important for an accurate thickness estimation. Indeed, when peaks or valleys are too steep in comparison to the fringe sampling, some phase jumps can be missed and a wrong absolute phase is recovered.

Furthermore, absolute phase estimation requires the assessment of a possible bias being unwrapped and real phase profile. This is usually done taking a reference point within the field of view. Here this reference point is given by the Newton black films that form during bubble inflation. The absolute thickness of these areas is half the illumination wavelength (in our case, 316 nm). In the frames where these are not present, we assumed that the process of film thinning is continuous and slow in comparison to the recording speed. At the end of the bubble’s life, i.e., when close to rupturing, this assumption is not necessary because black films are usually present. However, more complicated situations in which the estimation of a reference thickness may be more difficult can be addressed by adopting one of the variant systems proposed in the Discussion section and sketched in Figure S3.

Once the absolute phase map is obtained, the local thickness estimation is given by the formula: $s = \frac{\lambda }{{2\pi }}\frac{\phi }{{n - 1}}$, where *λ* is the illumination wavelength and *n* the refractive index of the solution. In this estimation *n* is considered a constant, which could induce some error when this is not true. In liquid films, of course, water evaporation alters the density of the solution and, in turn, the refractive index *n*. However, at the time scale of our experiments, we estimated that the error related to evaporation is negligible $(\delta s < 5\% )$ (see Supplementary Information).

It is well known that holographic measurements yield pseudo-3D images. This means that the measured thickness profile, *s*, is a projection on the image plane of the three-dimensional structure. However, the radial thickness, $\bar s$, i.e., the thickness along the normal to the bubble surface, can be retrieved by geometrical considerations (see Figure S6). Assuming that the upper and lower surface of the bubble are locally parallel, the relation between measured and radial thickness is $\bar s = s\sqrt {1 - \frac{{r^2}}{{R^2}}}$, where *r* is the distance from the center in the image plane and *R* is the radius. It is worth noting that in terms of the center, the two values are almost identical. For example, we estimated a relative error $\frac{{\bar s - s}}{s} = 1 - \sqrt {1 - \frac{{r^2}}{{R^2}}} < 1\% ,$ for $r < 1.3$ mm (see Figure S7). On the other hand, in proximity of the pipe’s border, the presence of meniscus deformation alters the estimation of $\bar s$ (see Figure S8). For this reason, we used *s* instead of $\bar s$ to calculate the volume or the draining rate at the borders because it conveys the same information with less geometrical assumptions (see Figure S9).

Herein, we used aqueous solutions that were sufficiently homogenous to not require any particular adjustment of the optical setup. However, when this is not the case and particularly inhomogeneous samples are to be studied, the use of two or more beams with different wavelengths is suggested^44^. Using different illuminating wavelengths with closer values gives us the possibility of creating a synthetic wavelength with a large value, and therefore we can enlarge continuous phase regions of the reconstructed wavefront. Often with this method, the unwrapping procedure is simplified or not required at all. Nonetheless, hologram registration with different light wavelengths results in a more complicated technique for both the hardware and software.

## Supplementary information

Supplementary Information

Thickness mapping during bubble growth

Bubble drainage

Bubble growth and drainage after 3d correction

Bubble growth (side view)

Fluid drainage

Complex fluid drainage

High-speed thickness mapping during bubble rupture
