# Supplementary material for: Quantitative imaging of the complexity in liquid bubbles’ evolution reveals the dynamics of film retraction
Source: Light Sci Appl. 2019 Jan 30;8:20. doi: 10.1038/s41377-019-0131-4 (PMC6351544; doi:10.1038/s41377-019-0131-4)
Supplement: Supplementary file 1 — Supplementary Information [file 41377_2019_131_MOESM1_ESM.docx]

**Quantitative imaging of the complexity in liquid bubbles’ evolution reveals the dynamics of film retraction**

Biagio Mandracchia^1,*^, Zhe Wang^1,2^, Vincenzo Ferraro^3^, Massimiliano Maria Villone^3^, Ernesto Di Maio^3^, Pier Luca Maffettone^3^, Pietro Ferraro^1^

*^1^CNR-ISASI, Istituto di Scienze Applicate e Sistemi Intelligenti «E. Caianiello» del CNR, Via Campi Flegrei 34, 80078 Pozzuoli, Napoli, Italy.*

*^2^College of Applied Sciences, Beijing University of Technology, 100124 Beijing, China.*

*^3^Dipartimento di Ingegneria Chimica, dei Materiali e della Produzione Industriale, Università di Napoli Federico II, Piazzale Tecchio 80, 80125 Napoli, Italy.*

**The dynamics and stability of thin liquid films have fascinated scientists over many decades. Thin film flows are central to numerous areas of engineering, geophysics, and biophysics and occur over a wide range of lengths, velocities, and liquid properties scales. In spite of many significant developments in this area, we still lack appropriate quantitative experimental tools with the spatial and temporal resolution necessary for a comprehensive study of film evolution. We propose tackling this problem with a holographic technique that combines quantitative phase imaging with a custom setup designed to form and manipulate bubbles. The results, gathered on a model aqueous polymeric solution, provide unparalleled insight into bubble dynamics through the combination of a full-field thickness estimation, three-dimensional imaging, and a fast acquisition time. The unprecedented level of detail offered by the proposed methodology will promote a deeper understanding of the underlying physics of thin film dynamics.**

***Keywords:*** *Metrology; Holography; Thin films, Thickness mapping.*

# Refractive index measurement

The phase signal obtained by DH is a measure of the optical path length experienced by the laser beam. This is related to the real space distance, z, covered by the light through the relation:

$\phi=\frac{2\pi n}{\lambda}z$ (1)

where n is the refractive index of the medium where the light is propagating.

To measure the actual thickness of the film, we have to evaluate the contribution to the optical path length due to the passage of light through the sample. This can be done using a hologram where no sample is present as a reference image. This image can be subtracted from the image of the sample and quantitative information about the thickness can be recovered:

$z_{s}=\frac{1}{n_{s}-n_{m}}\frac{\lambda}{2\pi}\phi$ (2)

$n_{s}$ is the refractive index of the sample and $n_{m}$ the refractive index of the medium. In order to evaluate the actual thickness of the film, we need to know, then, its refractive index.

We measured the refractive index of PA 5% solution using a square microfluidic channel of 200 μm thickness. We made two holographic maps of the channel: one with distilled water and the other with PA 5% solution. These were injected into the channel one after the other in order to look always at the same field of view, see Figure S2.

In the two phase images, the channel has the same profile but for a scaling factor due to the change in refractive index. Then, the refractive index of the solution could be obtained by the ratio:

$\frac{\phi_{s}}{\phi_{w}}=\frac{n_{s}-1}{n_{w}-1}$ (3)

During the experiments two solutions bunches of PA 5% were used. The measured values of refractive index were 1.47 ± 0.09 RIU and 1.73 ± 0.13 RIU, respectively.

# Numerical simulation of bubble growth

In order to give a plausible physical interpretation to the experimentally-observed non-monotonic trend of bubble thickness shown in Figure 2c of the main text, we have performed a Finite Element numerical simulation of a system mimicking the experimental one. An illustrative (not to scale) sketch of the computational domain is displayed in Figure S2a: we consider a cylindrical pipe with radius $R_{b}$ = 9 mm on the top of which, at time $t=0$, a flat film of PA solution with initial thickness $h_{0}$ = 25 μm is laid. To simulate imperfect film deposition, a liquid rim with height $h_{r0}$ = 1 mm is deposited on the edge of the pipe. Air is pumped from the bottom of the film with pressure $p_{i}$, making it inflate until reaching an almost hemispherical shape, then air pumping is stopped and the film morphological evolution under the action of gravity and surface tension is followed. An illustrative image of the bubble at a generic time instant during its inflation is reported in Figure S2b. For simplicity, we have considered an axisymmetric geometry (assuming also the liquid rim around the film to be axisymmetric), thus the computational domain is the 2D “slice” bounded by curves $\Gamma_{1}$, $\Gamma_{2}$, $\Gamma_{3}$, and $\Gamma_{4}$ in Figure S2a.

**Mathematical Model**. We have considered the system to be isothermal and the liquid incompressible. In addition, we have preliminarily evaluated the Ohnesorge number $\mathrm{Oh}=\mu/\sqrt{2\gamma\rho h_{0}}$. By taking the liquid viscosity $\mu\simeq$ 2.5 Pa s, the density $\rho$ = 1000 kg m^-3^, the surface tension $\gamma$ = 0.045 N m^-1^, and the film initial thickness $h_{0}$ = 25 μm, we have got $\mathrm{Oh}\simeq50\gg1$, thus we have neglected the effects of inertia. Hence, the film dynamics is governed by the mass and momentum balance equations in the Stokes formulation, reading

$\boldsymbol{\nabla}\cdot\boldsymbol{u}=0$ (S1)

$-\boldsymbol{\nabla}p\boldsymbol{+}\mu_{1}\nabla^{2}\boldsymbol{u}+\boldsymbol{\nabla}\cdot\boldsymbol{\tau+}\rho\boldsymbol{g}=\boldsymbol{0}$ (S2)

where $\boldsymbol{u}$, $p$, and $\boldsymbol{\tau}$ are the velocity, pressure, and viscoelastic stress tensor fields in the liquid (simulation outputs), $\boldsymbol{g}$ is the gravitational acceleration, and $\mu_{1}$ is the “Newtonian” contribution to the liquid viscosity.

It is known from the literature (1) that polymeric solutions such as the one considered un our experiments can be modeled through the Giesekus constitutive equation

$\lambda\overset{\nabla}{\boldsymbol{\tau}}+\boldsymbol{\tau+}\frac{\lambda\alpha}{\mu_{2}}\boldsymbol{\tau}^{2}=2\mu_{2}\boldsymbol{D}$ (S3)

with $\overset{\nabla}{\boldsymbol{\tau}}\boldsymbol{=}\frac{D\boldsymbol{\tau}}{Dt}\boldsymbol{-}\left( \boldsymbol{\nabla u} \right)^{T}\boldsymbol{\cdot\tau-\tau\cdot\nabla u}$ the upper-convected time derivative, $\lambda$ the viscoelastic liquid relaxation time, $\alpha$ the shear thinning parameter, $\mu_{2}$ the non-Newtonian contribution to the viscosity of the liquid, and $\boldsymbol{D}=(\boldsymbol{\nabla u+\nabla}\boldsymbol{u}^{T})/2$ the symmetric part of the velocity gradient tensor. The values of the rheological parameters $\mu_{1}$, $\mu_{2}$, $\alpha$, and $\lambda$ inputted in the simulations have been derived by fitting the experimental rheological for the PA solution used in the experiments and are $\mu_{1}$ = 0.5 Pa s, $\mu_{2}$ = 2.0 Pa s, $\alpha$ = 0.3, $\lambda$ = 0.5 s.

The balance equations that describe the system in Fig. S2a have been supplied with the following boundary conditions:

$\boldsymbol{T}\cdot\boldsymbol{n}=\gamma\boldsymbol{n}\nabla\cdot\boldsymbol{n+}p_{i}\boldsymbol{n}$ on $\Gamma_{1}$ (S4)

$\boldsymbol{u}=\boldsymbol{0}$ on $\Gamma_{2}$ (S5)

$\boldsymbol{T}\cdot\boldsymbol{n}=\gamma\boldsymbol{n}\nabla\cdot\boldsymbol{n}$ on $\Gamma_{3}$ (S6)

$\boldsymbol{u}\cdot\boldsymbol{n}=0$ on $\Gamma_{4}$ (S7)

$\left( \boldsymbol{I}-\boldsymbol{nn} \right)\cdot\left( \boldsymbol{T}\cdot\boldsymbol{n} \right)=\boldsymbol{0}$ on $\Gamma_{4}$ (S8)

Equation S4, where $\boldsymbol{T}=-p\boldsymbol{I}+2\mu_{1}\boldsymbol{D}+\boldsymbol{\tau}$ is the total stress tensor in the fluid and $\boldsymbol{n}$ is the outwardly directed unit vector normal to the boundary, is the Young-Laplace condition on the film bottom face with an extra-contribution given by the inflation pressure $p_{i}$. Equation S5 is the adherence condition between the liquid film and the solid edge of the cylindrical pipe. Equation S6 is the Young-Laplace boundary condition on the film top face. Finally, Equations S7 and S8 express the system axial symmetry.

Since the film has no inertia, no initial condition on the liquid velocity was needed, whereas, for what concerns the stress, we have assumed that, prior to the beginning of inflation, the sheet was stress-free, namely, $\boldsymbol{\tau}=\boldsymbol{0}$.

**Numerical Technique**. The mass balance, the momentum balance, and the constitutive equations reported above have been solved through the Finite Element Method (FEM) with an Arbitrary Lagrangian Eulerian (ALE) formulation. The numerical code uses stabilization techniques widely described in the literature, such as SUPG and log-conformation (2-4). A detailed description of the algorithm employed to track the film surface is given in (5). As apparent in Figure S2a, the system has a symmetry axis coinciding with the $z$-axis, thus the physical domain could be reduced to a 2D axisymmetric computational domain. The latter has been discretized by an unstructured mesh made of triangular elements. During the simulation, the film deforms, making the mesh elements progressively deform. Every time the mesh quality went below a threshold, a re-meshing has been done and the computed velocity, pressure, and stress fields have been projected from the old mesh to the new one (6,7). Preliminary convergence tests have been performed in space and time, i.e., mesh resolution and time step for the numerical solution of the model equations have been selected such that invariance of the results upon further refinements has been ensured. Second order time integration has been used.

# Estimation of the thickness normal to the bubble surface

It is well known that holographic measurements yield pseudo-3D images. This means that the measured thickness profile is a projection on the image plane of the three-dimensional one. However, the radial thickness, i.e. the thickness along normal to the bubble surface, can be retrieved by geometrical considerations, see Figure S3. Assuming that the upper and lower surface of the bubble are spherical and locally parallel, it is possible to calculate the radial thickness from the measured one using the relation $\bar{s}=scos( \frac{\pi}{2}-\alpha)=s\sin\alpha$. The relative error is $\frac{\bar{s}-s}{s}=1-\sin\alpha$ where $\sin\alpha$ can be replaced as a function of the radial position on the image plane using $r=R\cos\alpha$ from witch $\sin\alpha=\sqrt{1-\frac{r^{2}}{R^{2}}}$ . Replacing $\sin\alpha$ in the formula of the relative error found before $\frac{\bar{s}-s}{s}=1-\sqrt{1-\frac{r^{2}}{R^{2}}}$, see Figure S4. For example, we estimated a relative error $\frac{\bar{s}-s}{s}=1-\sqrt{1-\frac{r^{2}}{R^{2}}}<1\%,$ for $r<1.3$mm.

However, the bubble’s shape deviates from the spherical geometry in proximity of the pipe’s border. Here, the presence of meniscus deformation modifies the estimation of the radial thickness along the borders, see Figure S5.

# Volume calculation

The estimation of the entire volume of the film was calculated as a sum of the parallelepipeds witch have got the pixels area as base and the measured thickness as height $Vol=\sum\Delta x_{i}\Delta y_{i}\Delta s_{i}$. The calculation of the volume is done using the measured thickness because the radial one in not well estimated along the pipe’s border due to the meniscus, see Figure S6.

# Water evaporation estimation

To evaluate all possibilities, we estimate also the effect of water evaporation on the refractive index during the experiments using $\frac{n^{2}-1}{n^{2}+2}=\rho R(\lambda)$, where $n$ is the refractive index. The velocity of water evaporation is $3*{10}^{-8}(g/{mm^{2}})/s$. After 3.6s, the density of the solution in a circle of $1 mm^{2}$ area on top of the bubble increments by 0.5%, which leads to a 1% increment of the refractive index. Thus, the relative error on thickness estimation due to water evaporation is:

$$\frac{\delta s}{s}=\frac{\Delta n/n}{(n-1)/n+\Delta n/n}=0.03$$

For $n=1.47$ and ∆n=0.0147. Hence, we can argue that at the time scales of our experiments the effect of water evaporation, and the related change of the refractive index along the film, are negligible.

# References

1. Larson, R. G. Constitutive Equations for Polymer Solutions and Melts, Butterworths, Stoneham 1988.

2. Guénette, R.; Fortin, M. A new mixed finite element method for computing viscoelastic flows. J. Non-Newton. Fluid Mech. 1995, 60, 27–52.

3. Bogaerds, A. C.; Grillet, A. M.; Peters, G. W.; Baaijens, F. P. Stability analysis of polymer shear flows using the extended pom–pom constitutive equations. J. Non-Newton. Fluid Mech. 2002, 108, 187–208.

4. Brooks, A. N.; Hughes, T. J. Streamline upwind/Petrov–Galerkin formulations for convection dominated flows with particular emphasis on the incompressible Navier–Stokes equations. Comp. Meth. Appl. Mech. Eng. 1982, 32, 199–259.

5. Villone, M. M.; Hulsen, M. A.; Anderson, P. D.; Maffettone, P. L. Simulations of deformable systems in fluids under shear flow using an arbitrary Lagrangian Eulerian technique. Comput. Fluids 2014, 90, 88–100.

6. Hu, H.H.; Patankar, N. A.; Zhu, M. Y. Direct numerical simulations of fluid–solid systems using the arbitrary Lagrangian–Eulerian technique. J. Comput. Phys. 2001, 169, 427–462.

7. Jaensson, N. O.; Hulsen, M. A.; Anderson, P. D. Stokes–Cahn–Hilliard formulations and simulations of two-phase flows with suspended rigid particles. Comput. Fluids 2015, 111, 1–17.


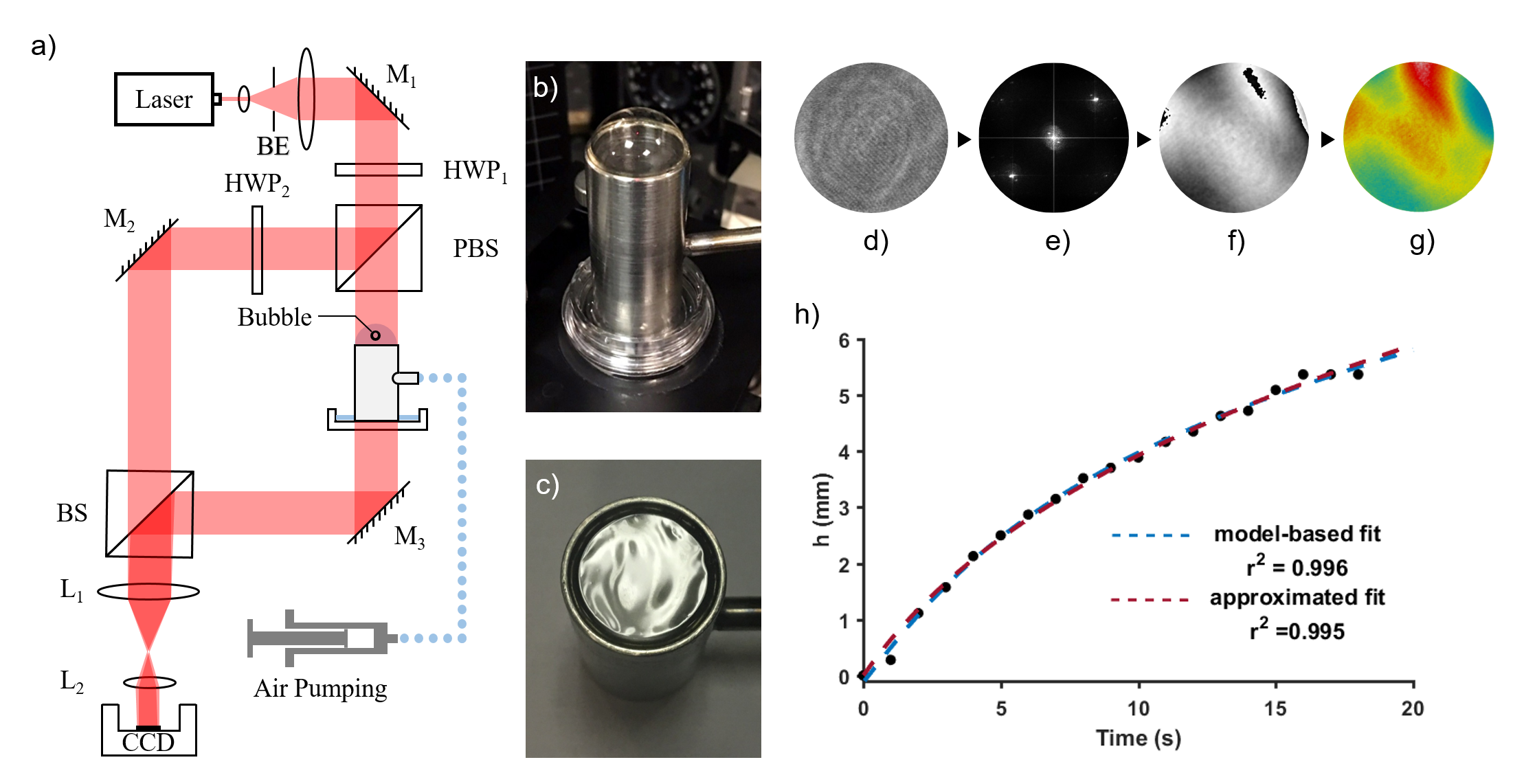


**Figure S1:** **Holographic Setup for thin liquid film measurement.** a) Sketch of the experimental setup. BE: beam expander; M: mirror; HWP: half wavelength plate; PBS polarizing beam slitter; BS: beam splitter; L: lens. b) Side view of the metal pipe with a grown bubble on it. c) Top view of the pipe with the polymeric film on it before blowing. d-g) Depiction of the processing of digital holograms: recorded hologram (d), Fourier spectrum of the hologram (e), wrapped phase map of the object (f), final quantitative phase image of the object (g). h) The height, h, of the center of the bubble as function of the time with a constant in-flow of 0.015ml/s.


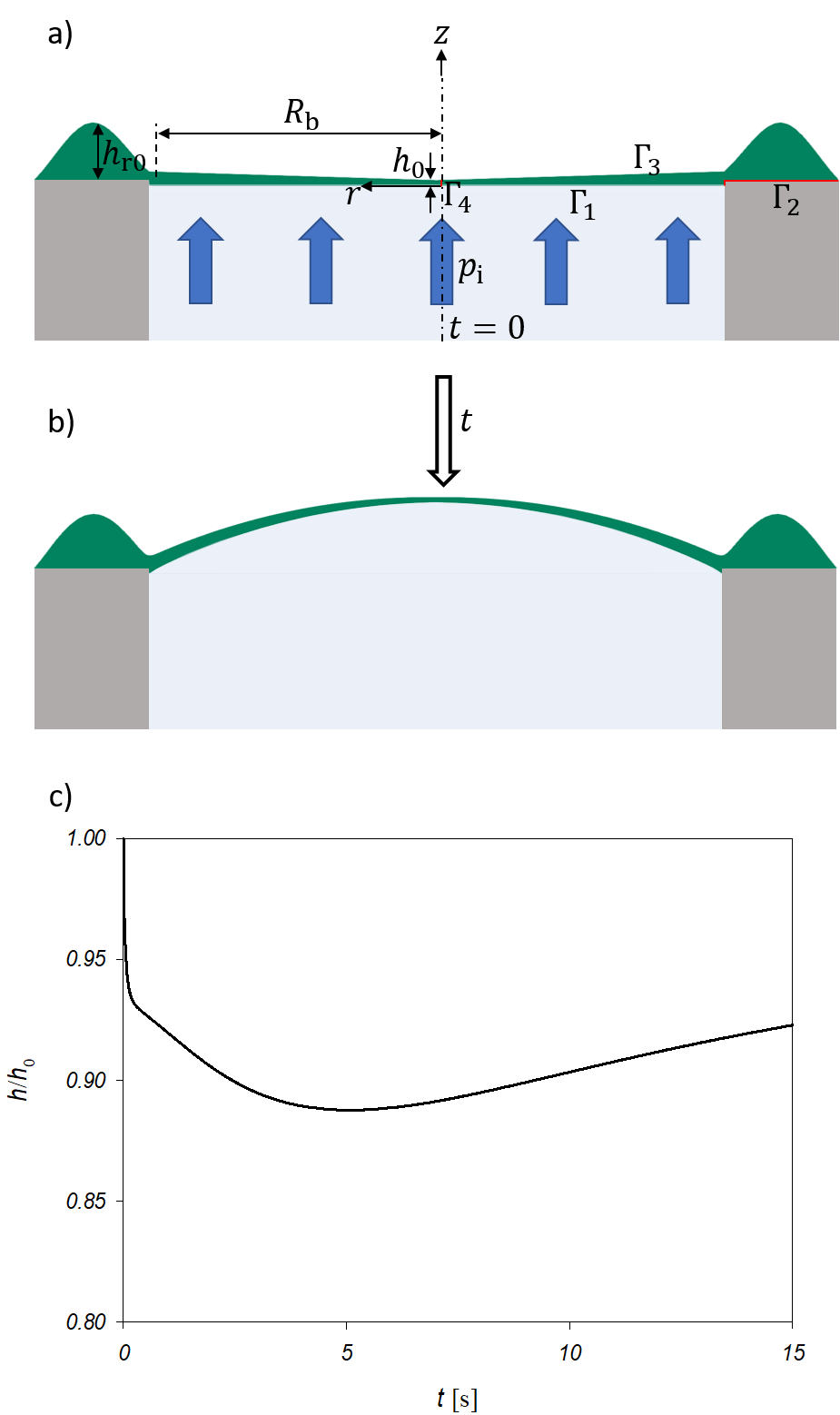


**Figure S2: Numerical Simulation.** (**a**) Sketch of the geometry of the computational domain at time *t* = 0. (**b**) Sketch of the geometry of the computational domain at a generic time instant during bubble inflation. (**c**) Numerical temporal trend of the thickness at the center of the film *h* normalized by its initial value *h*_0_.


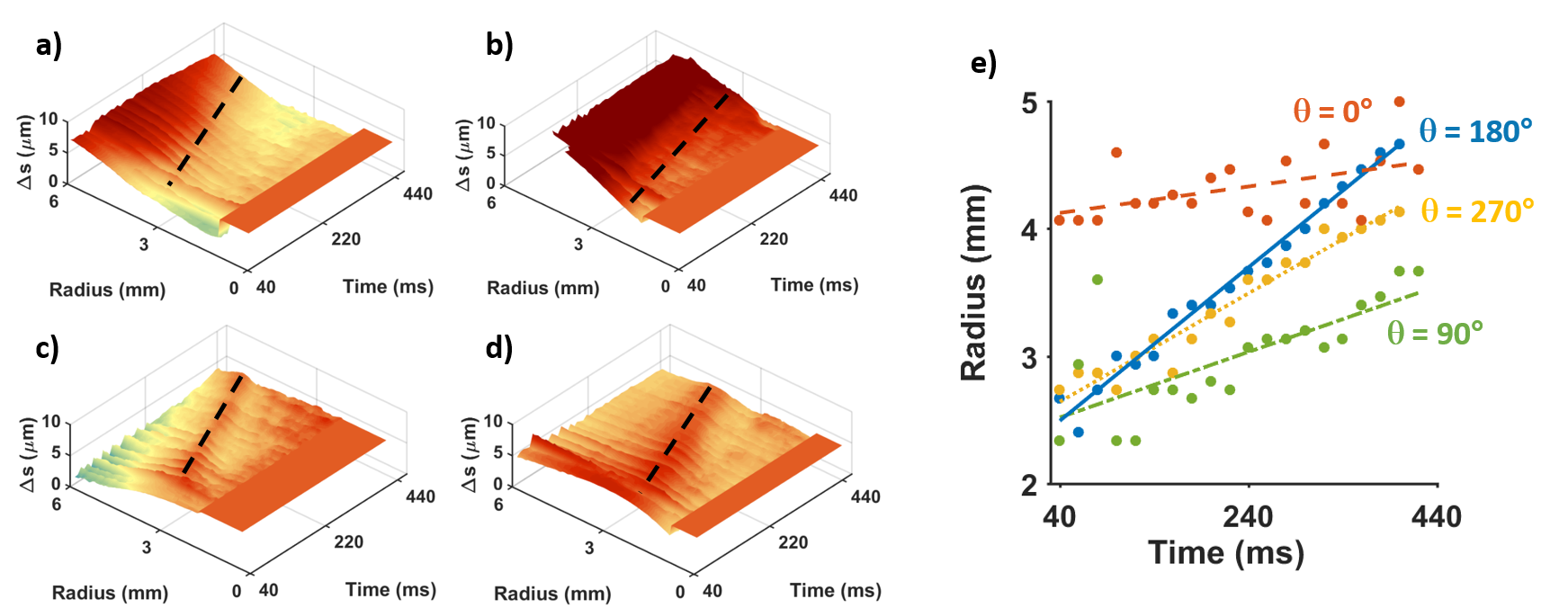


**Figure S3: Expansion of the black film during bubble rupture.** (a-d) Evolution of the black spot in four different directions: θ = 0°, 90°, 180°, and 270°, respectively. (e) The rim of the black film expands faster along the directions θ = 180° and 270°, in correspondence of thinner regions. The residual errors expected for PUMA unwrapping are smaller than the marker size.

**
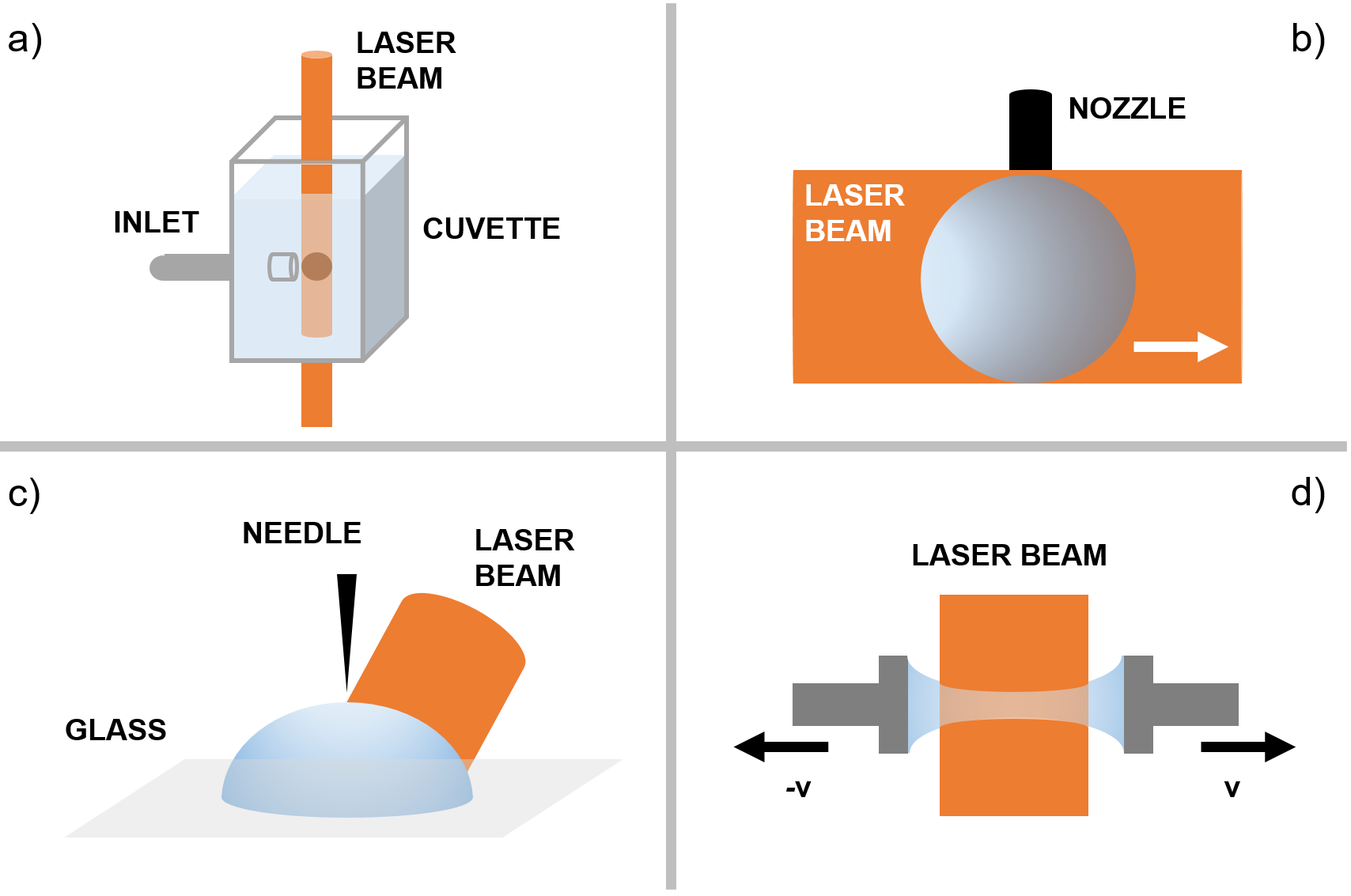
Figure S4: Alternative implementations of DH for thin film evolution dynamic study.** (**a**) the metal pipe can be replaced by a cuvette. (**b**) The object beam can image a bubble hanging from a nozzle. (**c**) The bubble can be grown on top of a glass surface. (**d**) A diaphragm can be used to grow and stretch a flat film.


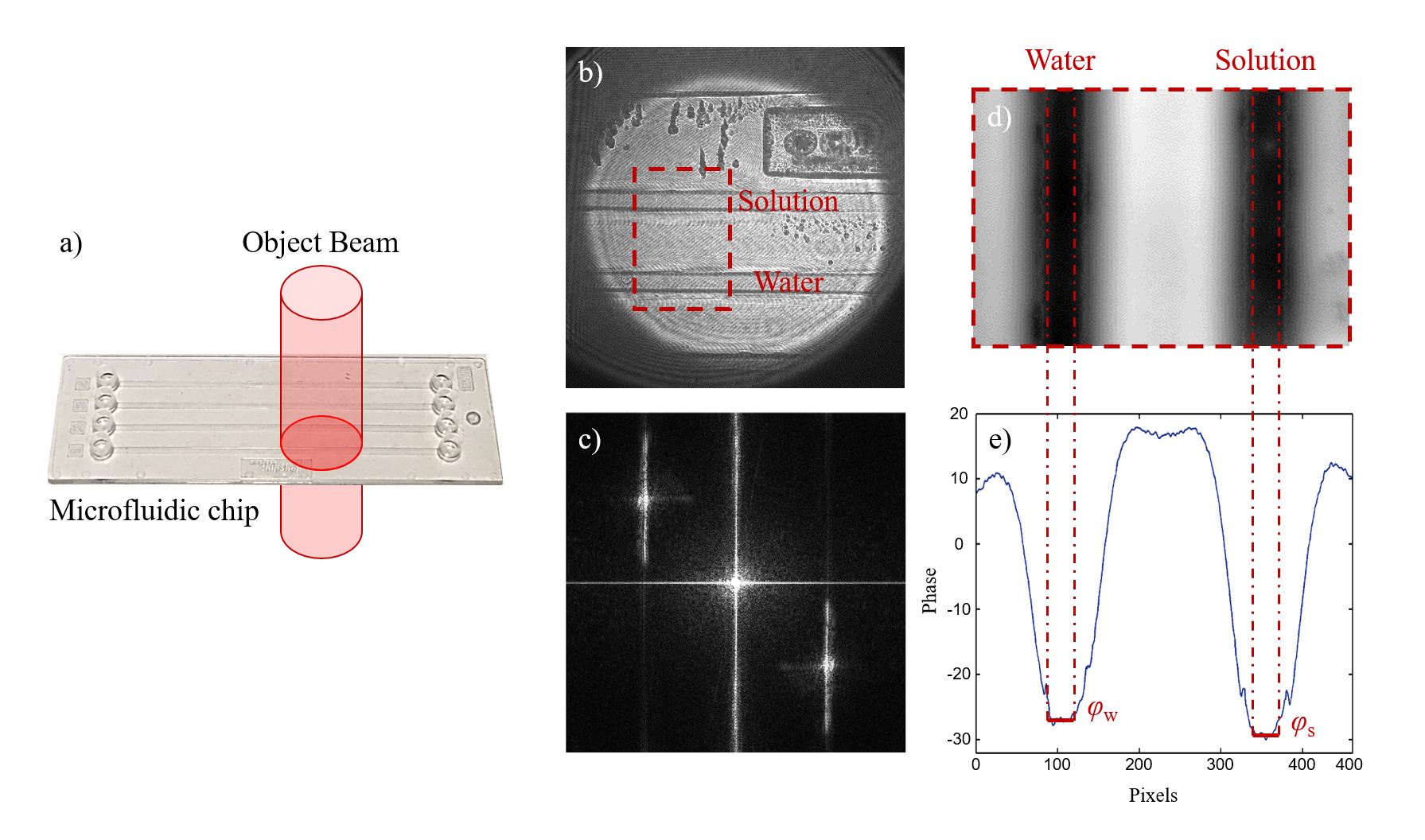


**Figure S5: Refractive index measurement.** a) sketch of the microfluidic chip used for the assessment of the refractive index. b) Hologram of two microfluidic channels containing water and the PA %5 solution, respectively. c) Fourier transform of the hologram in b). d) Reconstructed phase map and e) average phase profile.


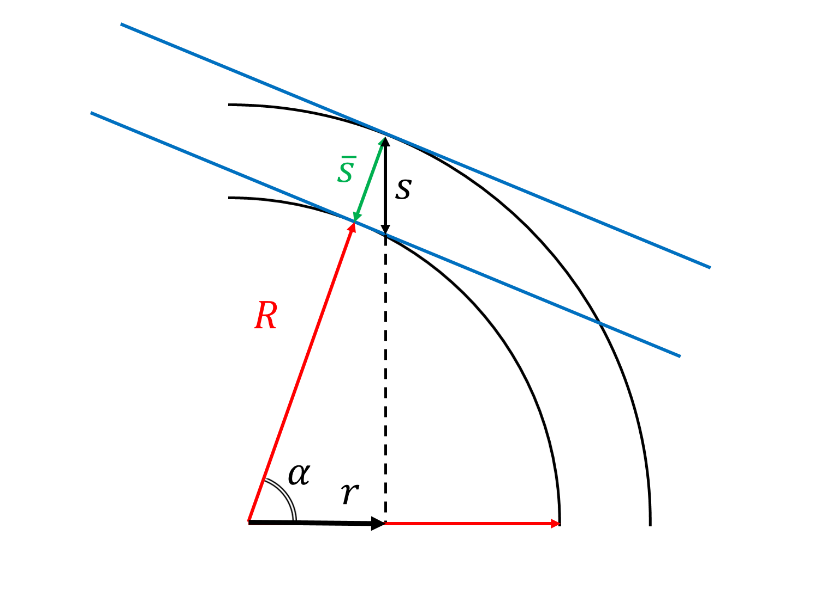


**Figure S6**: **Film thickness evaluation along the radial direction.** Scheme of the thickness measurement, as projected on the image plane, $s$, and the true thickness normal to the bubble surface, $\bar{s}$. By knowing the radius of the bubble, *R*, and *α*, it is possible to retrieve $\bar{s}$ from $s$.


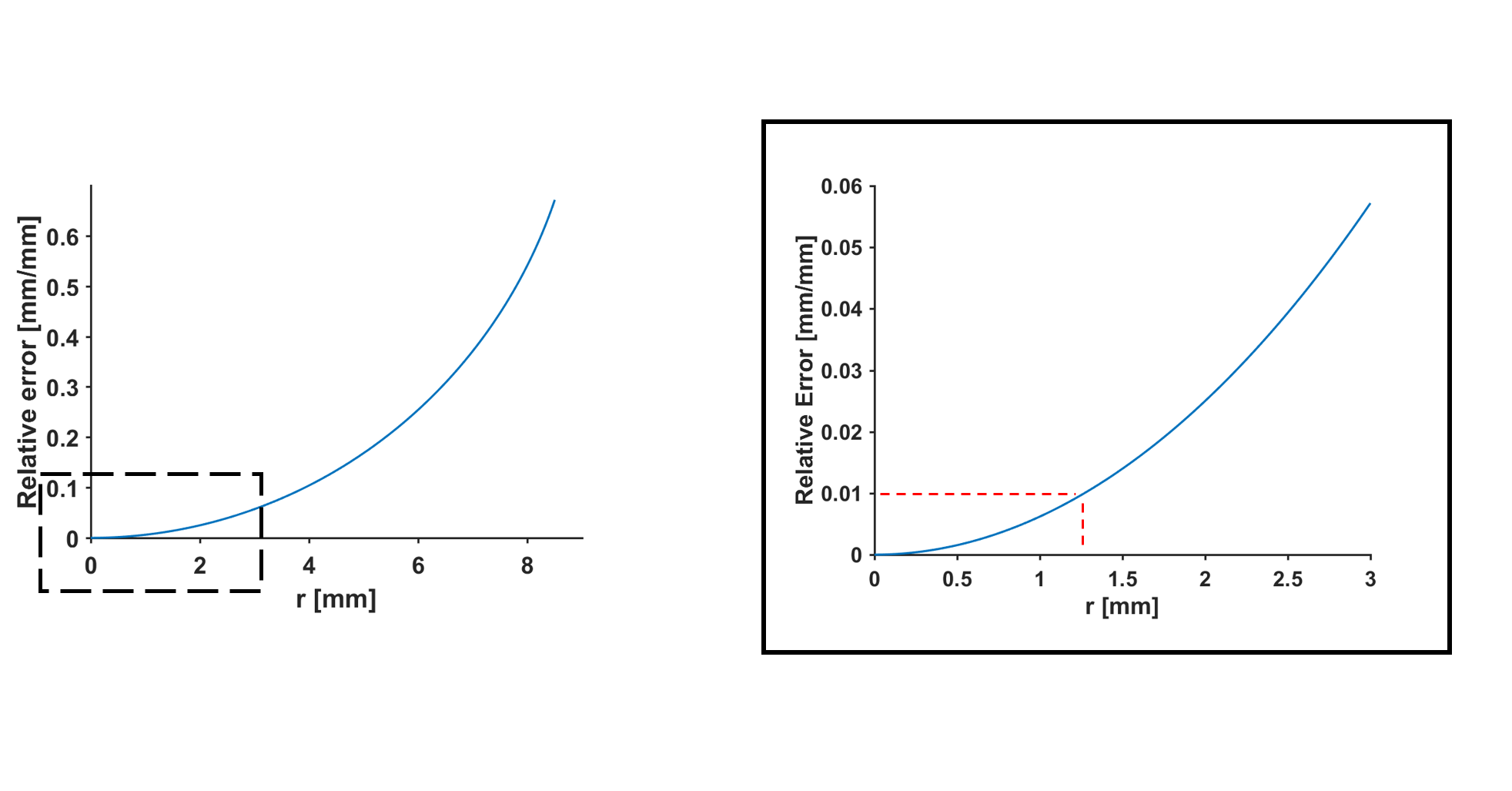


**Figure S7**: **Relative error as a function of the basal radius.** Plot of the relative error as function of the basal radius of the bubble calculated for all the sample (left) and only the center (right).


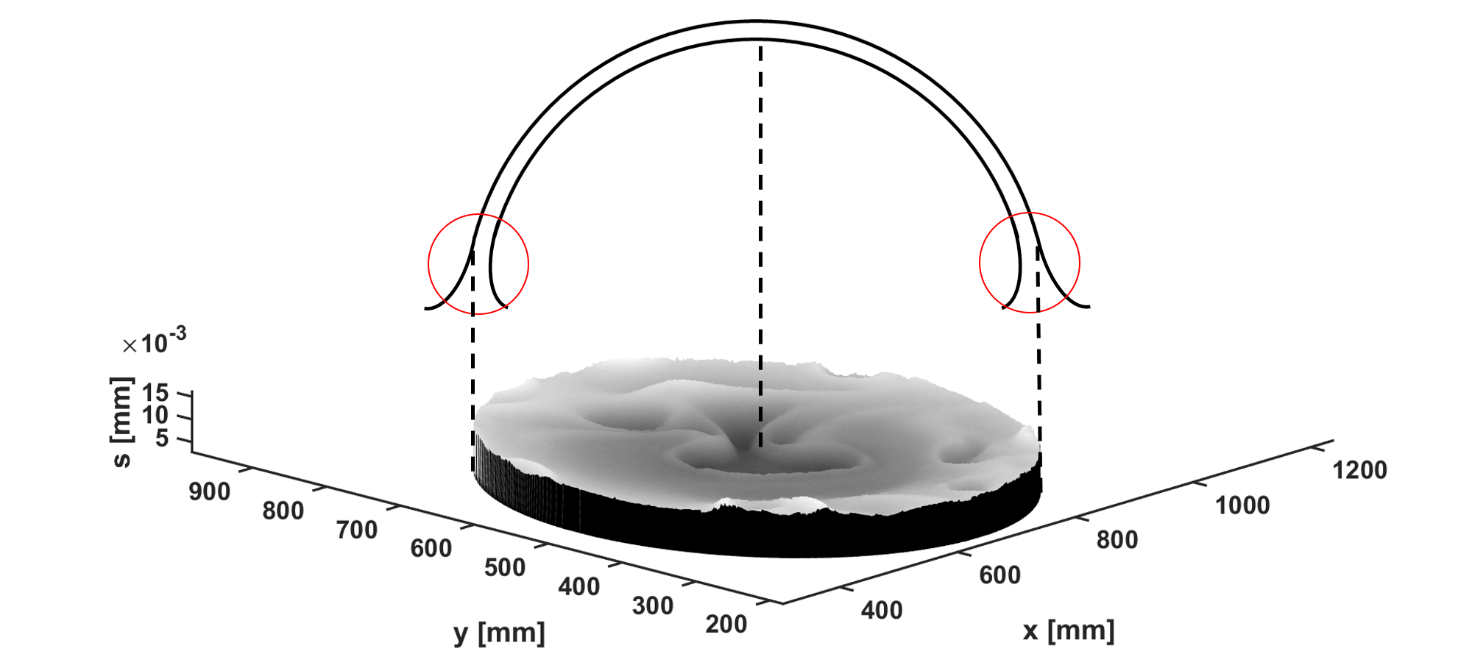


**Figure S8**: **Measured thicknesses of the bubble projected on the image plane.** In the red circles, the meniscus deformations close to the border of the pipe are evidenced. Below, the behavior of the thickness as a function of the coordinates $(x_{i},y_{i})$.


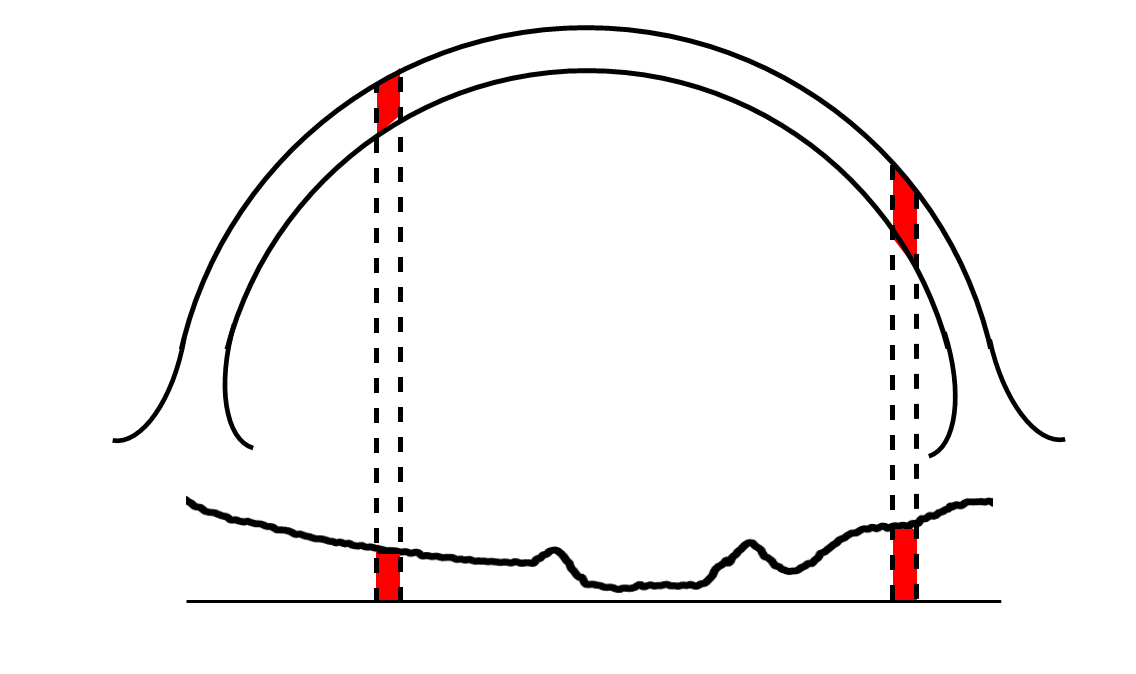


**Figure S9**: **Pseudo 3D holographic imaging.** Depiction of the holographic thickness measurement as a projection of the three-dimensional profile.
